# Polymeric biomaterials for wound healing

**DOI:** 10.3389/fbioe.2023.1136077

**Published:** 2023-07-27

**Authors:** Cristiana Oliveira, Diana Sousa, José A. Teixeira, Pedro Ferreira-Santos, Claudia M. Botelho

**Affiliations:** ^1^ CEB—Centre of Biological Engineering, University of Minho, Campus de Gualtar, Braga, Portugal; ^2^ LABBELS—Associate Laboratory, Braga, Portugal; ^3^ Department of Chemical Engineering, Faculty of Science, University of Vigo, Ourense, Spain

**Keywords:** wound healing, polymeric biomaterials, skin regeneration, natural materials, health

## Abstract

Skin indicates a person’s state of health and is so important that it influences a person’s emotional and psychological behavior. In this context, the effective treatment of wounds is a major concern, since several conventional wound healing materials have not been able to provide adequate healing, often leading to scar formation. Hence, the development of innovative biomaterials for wound healing is essential. Natural and synthetic polymers are used extensively for wound dressings and scaffold production. Both natural and synthetic polymers have beneficial properties and limitations, so they are often used in combination to overcome overcome their individual limitations. The use of different polymers in the production of biomaterials has proven to be a promising alternative for the treatment of wounds, as their capacity to accelerate the healing process has been demonstrated in many studies. Thus, this work focuses on describing several currently commercially available solutions used for the management of skin wounds, such as polymeric biomaterials for skin substitutes. New directions, strategies, and innovative technologies for the design of polymeric biomaterials are also addressed, providing solutions for deep burns, personalized care and faster healing.

## 1 Introduction

Skin is the first biological barrier against several invaders, such as external biological, physical, and chemical agents, and radiation. This organ has various important roles: body temperature regulation and vitamin D production. Moreover, the skin functions as a scaffold for other tissues and organs such as hair follicles (Hazrati et al., 2022). Given the clear importance of skin, not only in physical health, but in people’s mental health and social behaviour, it is crucial that this tissue is well cared for. Skin is often subject to injury, so effective wound healing is essential.

**FIGURE 1:**
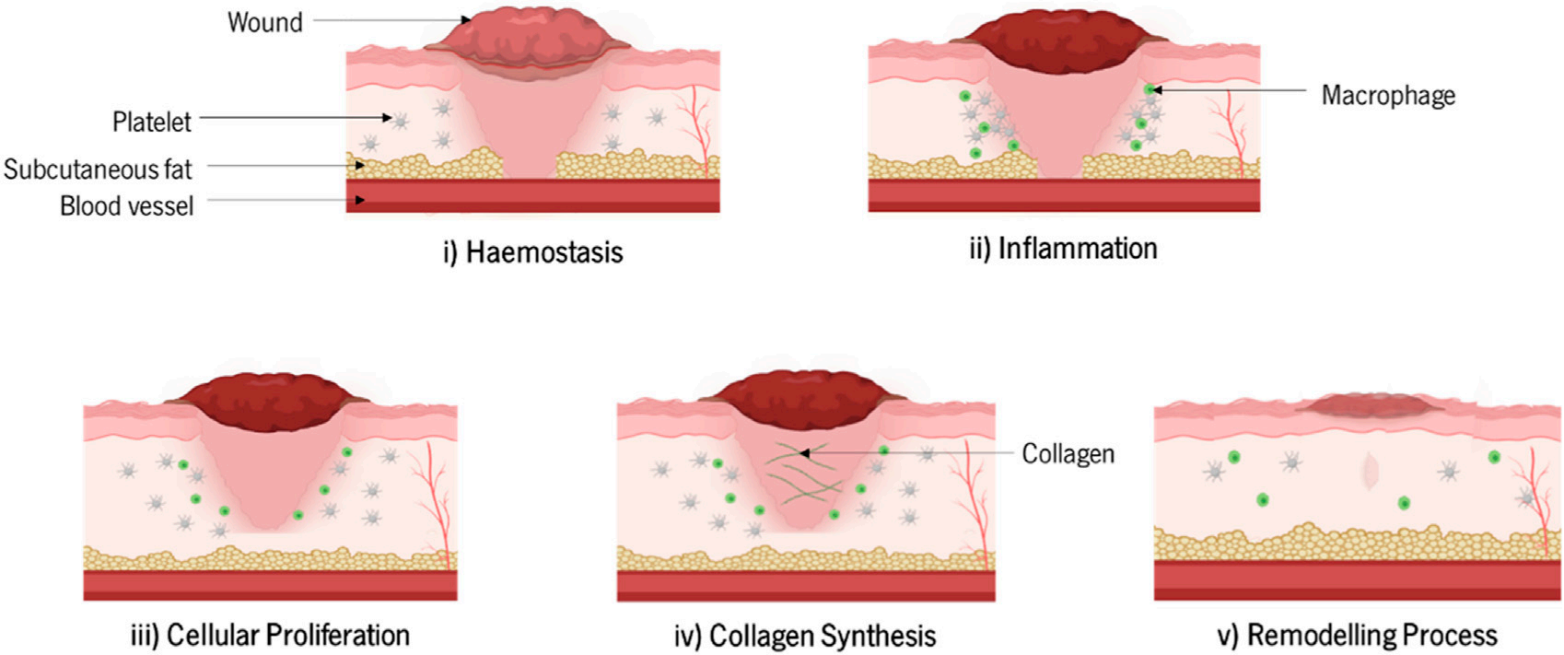
Stages of the wound healing process.

Wound healing is the biological process of tissue repair, which includes a sequence of cellular events that at a given time lead to new healthy tissue (Suamte et al., 2023), (Ghosh and Gaba, 2013) (Figure 1). This sequence of events comprises i) hemostasis, which consists of the restriction of the microvasculature system, platelets aggregation, degranulation, and fibrin formation, ii) inflammation, when neutrophil, monocyte, and lymphocyte infiltration and macrophage differentiation occur, iii) cellular proliferation, comprising mesenchymal cell differentiation/migration to the wound site, re-epithelialization, and angiogenesis, iv) collagen synthesis and extracellular matrix formation (ECM), and v) remodeling process, including the maturation of vascular functions and ECM remodeling (Hazrati et al., 2022), (Guo and Dipietro, 2010), (Thomas Hess, 2011). Hemostasis and chemotaxis seem to be the first evidence of the inflammatory phase. White blood cells and thrombocytes equally generate mediators and cytokines, contributing to the inflammatory response. In addition to the platelet growth factor, which is responsible for collagen destruction, fibroblasts attraction, new blood vessel formation, and re-epithelialization, mediators such as serotonin and histamine, are released from platelets to enhance the cell permeability. In turn, fibroblasts settle on fibrin and proliferate, beginning to synthesize collagen and some glycosaminoglycans. This step allows wound stabilization and the completion of the wound healing process through the migration of pre-existing cells or the circulation of stem/progenitor cells to the damaged site and their differentiation into epithelial cells. This step is followed by the maturation of the vascular function and ECM remodeling (Hazrati et al., 2022), (Eming et al., 2007).

Wounds can be classified as acute or chronic, depending on the type and magnitude of the skin injury. In this way, small wounds consisting of superficial-partial thickness skin defects are classified as acute wounds and are re-established by a simple contraction along with cell growth in the wound area. On the other hand, large skin wounds consisting of deep-partial and full-thickness defects require a longer time for healing. If the healing process is halted, due to an arrest at the inflammation stage and/or infection for example, the wound becomes chronic (Mogoşanu and Grumezescu, 2014), (Hosseini and Shafiee, 2021). The development of chronic wounds may be triggered by malnutrition, kidney infections, immunodeficiency, diabetes, and age, among other factors (Mogoşanu and Grumezescu, 2014), (Nyame et al., 2015).

Several biological phenomena can occur, such as fibroplasia, granulation, contraction, epithelialization, and scar maturation, leading to significant scaring and poor diagnosis for the patient (Hazrati et al., 2022). Therefore, the need to develop better systems to mimic wound healing physiology is of upmost importance. Polymer-based biomaterials can be used for wound treatment, as wound dressings and regenerative scaffolds. Indeed, polymers have an important feature, as they can form hydrogels, which can absorb and release water, being therefore effective for controlling excessive wound exudates, while keeping a moist environment at the wound site enabling healing (Kohli et al., 2019), (ter Horst et al., 2019).

This review paper aims to describe several solutions used for the management of skin wounds, such as polymeric biomaterials for skin substitutes. Moreover, this research focuses on comparing and discussing the advantages and disadvantages of currently commercially available distinct natural and synthetic polymers used in cutaneous tissue engineering. New directions, strategies and innovative technologies for the design of polymeric biomaterials are also addressed, providing solutions for deep burns, personalized care, and faster healing.

## 2 Polymeric-based biomaterials for wound healing

Materials to be used for wound treatment must have biocompatibility, like chitosan (Liu, 2018). Polymers have interesting properties. They are easily modified through chemical processes, with the ability to form 3D structures for scaffolds and tailor their surface functionality (Paul and Sharma, 2004). Polymers flexibility in terms of topology, dimensions, and chemistry make them suitable to act as a drug delivery system enhancing wound repair. Therefore, polymers are great candidates to be used as skin substitutes (Talikowska et al., 2019), (Negut et al., 2020). Table 1 describes the application of different polymers for wound healing and skin applications.

**TABLE 1:** Polymers commonly employed as scaffolds for wound healing and skin tissue engineering applications.

Polymers	Loaded items	Application	References
Poly(ε-caprolactone)	Usnic acid	Full-thickness wounds	Chandika et al. (2022)
Chitosan/Gelatin	Silver nanoparticles/Phosphotungstic acid-polydopamine nano-flowers	Wound healing	Zhou et al. (2022)
Collagen/Hydroxypropyl methylcellulose	Povidone-iodide	Regenerative tissue engineering	kumar Kesavan et al. (2022)
Chitosan/Polyvinyl alcohol/Polyvinylpyrrolidone/Maltodextrin	*Satureja mutica*/*Oliveria decumbens* essential oil	Wound dressing	Barzegar et al. (2021)
Zein/Polycaprolactone/Collagen	Zinc oxide nanoparticles/Aloe-vera	Wound healing	Ghorbani et al. (2020)
Collagen	Polydatin	Chronic non-healing wounds	Selvakumar and Lonchin (2022)
Collagen/Poly-d-l-lactide–glycolide	Glucophage	Diabetic wounds	Lee et al. (2015)
Sodium alginate/Gelatine	Paeoniflorin	Diabetic wounds	Yu et al. (2022a)
Calcium alginate/Polycaprolactone/Gelatine	Coconut oil	Wound healing	Mohamadi et al. (2022)
Chitosan/Gelatine	Platelet-rich plasma	Chronic wounds	Koyuncu et al. (2022)
Fibrin/Chitosan/Keratin	Ferulic acid loaded silica microspheres	Chronic and infected wounds	Sivakumar et al. (2021)
Polyethylene glycol diacrylate/Catechol-modified hyaluronic acid	Ag-doped mesoporous silica nanoparticles	Wound dressing	Huang et al. (2023)
Thiolate hyaluronic acid/Silk fibroin	Bioactive glass nanoparticles	Wound healing	Yu et al. (2022b)
Polyurethane/Chitosan	Linezolid	Diabetic wounds	Teaima et al. (2022)

### 2.1 Natural polymers

Natural polymers, as the name states, are obtained from natural sources such as microbial, vegetable, and animal biomass. The characteristics of these polymers, namely biodegradability, biocompatibility, and biological activity, make them ideal for health-related applications (Negut et al., 2020). Moreover, natural-derived polymers can be used to replace natural ECM structural components and skin cellular background (Negut et al., 2020), (Portela et al., 2019). Indeed, these biopolymers are an asset for the design of versatile materials, as they meet the requirements for tissue engineering applications. When subjected to enzymatic degradation, natural polymers form by-products with low toxicity, which in most cases are well accepted by living organisms. However, natural polymers have some limitations, namely the difficulty to control their degradation rates/processes (Negut et al., 2020), (Bao et al., 2009), (Sill and von Recum, 2008). Regarding wound healing applications, natural polymers can be used as bioactive materials enhancing regeneration, as vehicles for drug delivery, and as scaffold formations with 3D networks promoting local tissue regeneration. When selecting polymeric materials for biomedical applications, several characteristics must be considered, including architecture, solubility, pore size, degradability, water absorption capacity, and electrical charge (ter Horst et al., 2019).

In the following sub-sections, several types of natural biopolymers already used in regenerative medicine processes will be discussed.

#### 2.1.1 Alginate

Alginate has been used for skin regeneration for several years (Barbu, 2021). Alginate is a linear anionic polymer consisting of mannuronic acid (M blocks) and guluronic acid (G blocks) units. Alginate can be obtained from some bacteria, brown algae and kelp (ref). Alginate is a versatile polymer, that can form hydrogels and its mechanical properties can be easily adapted by the type and concentration of a cross-linker (ter Horst et al., 2019), (Augst et al., 2006). Alginate has several advantageous properties, such as biocompatibility, biodegradability, low-immunogenicity, simple gelation, ability to control its degradation, and relatively low cost (Suamte et al., 2023), (ter Horst et al., 2019), (Ceccaldi et al., 2017). When this polymer has a higher ratio of G blocks it is easier to process and it seems to have lower immunogenicity (ter Horst et al., 2019), (Aravamudhan et al., 2014). This polymer also has antiseptic properties, low toxicity, conformability, good water absorption and optimal water vapor transmission rate, which are some of the main characteristics required for wound dressings (Suamte et al., 2023), (Barnett and Varley, 1987). Given its beneficial properties, alginate can also be used in the creation of scaffolds for tissue engineering. Indeed, scaffolds consisting of alginate microspheres, alginate-chitosan, chito-oligosaccharides (COS) with collagen (alginate–chitosan–COS–collagen) and a combination of alginate, chitosan and COS (alginate–chitosan–COS) have been applied as a skin substitute, revealing improved biocompatibility and replicating the skin microenvironment (Suamte et al., 2023), (Chandika et al., 2015). Mobaraki et al. (2021) developed a drug delivery scaffold by conjugating alginate and collagen and incorporated curcumin nanoparticles to enhance wound healing properties. The results showed that wound healing increased by over 90% within 14 days. This study also demonstrated the ability of an alginate/collagen scaffold to incorporate curcumin nanoparticles and consequently improve skin healing. Gao et al. (2022) developed an innovative 3D culture system using an alginate microsphere-collagen hydrogel, which demonstrated the ability to enhance human umbilical cord mesenchymal stem cell survival, allowing their sustained release to boost wound healing.

#### 2.1.2 Cellulose

Cellulose is an abundant polymer in nature and can be obtained from plants (vascular plants or algae) and bacteria (*Acetobacter xylinum*) (ter Horst et al., 2019). This polymer offers a stable matrix necessary for tissue engineering purposes and is available in diverse forms with exceptional biocompatibility, as is the case of viscose cellulose sponge (Suamte et al., 2023), (Märtson et al., 1998).

Bacterial cellulose has an interesting feature, a porous structure similar to skin. It is a highly hydrophilic material, which is a very important feature for a wound dressing, as a wound requires a moist environment (Kucińska-Lipka et al., 2015). Diaz-Gomez et al. (2022) developed a 3D printed carboxymethyl cellulose scaffold loaded with platelet rich plasma for the treatment of diabetic wounds. This scaffold revealed a sustained release of growth factors and boosted angiogenesis, granulation, and re-epithelialization in skin defects, highlighting its suitability to act as an active dressing for patients with diabetes (Diaz-Gomez et al., 2022). Cellulose can also be associated with different components to further enhance its effect, as demonstrated by Alavi and Nokhodchi (2020). The authors combined cellulose with zinc oxide nanoparticles and reported that this combination improves the mechanical and anti-bacterial properties of the wound dressing. Additionally, Madub et al. (2021) reported the possibility to prepare a nanofibrous scaffold using ulvan cellulose. Using an in vitro assay with fibroblast cells, the study demonstrated that the scaffold enhanced cell growth and accelerated angiogenesis without foreign body response in an in vitro biocompatibility test. Xu et al. (2022) prepared a chitosan-cellulose hydrogel dressing integrating microspheres for wound healing. This hydrogel exhibited good drug release performance, improved antibacterial activity, and had the ability to support the survival and proliferation of human adipose-derived stem cells. The main limitation of this natural polymer is the high cost associated with its purification process.

#### 2.1.3 Chitosan/chitin

Chitosan is a polysaccharide obtained from the deacetylation of chitin. Chitin is the structural element crustaceans or fungi exoskeleton (ter Horst et al., 2019), (Ahmadi et al., 2015). The main limitation of chitosan/chitin is its poor stability. As it is a weak base, chitosan/chitin is soluble in an acidic solution, but is not soluble in a neutral solution (ter Horst et al., 2019). This polymer has several beneficial properties, such as homeostasis, mucoadhesion, hydrophilicity and non-toxicity. Its similarity to the ECM structure improves cell adhesion and proliferation, making chitosan/chitin an auspicious candidate for tissue engineering applications (Suamte et al., 2023), (Amado et al., 2008). Indeed, studies have shown that chitosan can improve the function of inflammatory cells during wound healing (Feng et al., 2021). Moreover, it has been reported that a chitosan coating enhances the biocompatibility and hydrophilicity of different biomaterials (Kweon et al., 2003), (Shalumon et al., 2011). Fujita et al. (2004) developed a chitosan hydrogel cross-linked by UV light irradiation that revealed resistance against *Escherichia coli* growth. Dacron grafts combined with this chitosan hydrogel demonstrated antibacterial activity with considerable local infection inhibition. A study with diabetic rats demonstrated that a chitosan/collagen scaffold fused with Thymosin beta-4 (TB4) presented a sustained release of TB4 leading to faster proliferation and migration of glucose-treated human umbilical vein endothelial cells with heightened angiogenesis (Ti et al., 2014). Rezaii *et al.* also prepared a chitosan-collagen scaffold with the incorporation of curcumin nanoparticles. This scaffold led to the successful healing of full-thickness punch wounds on a Wistar rat model (Rezaii et al., 2019). The developed scaffold revealed an excellent wound-healing potential since it allowed new blood vessel formation and increased collagen production. Additionally it led to changes at the wound site regarding the granulation tissue density and epidermis thickness (Rezaii et al., 2019). A porous 3D scaffold composed of chitosan hydrochloride, collagen, carboxymethyl cellulose and β-glycerophosphate presented water absorption capacity and sustained released of rosuvastatin. Maged et al. also reported that the loading of mesenchymal stem cells into the scaffold further enhanced fibroblast proliferation and skin regeneration in a wound-healing model of Albino rats (Maged et al., 2019). Vakilian et al. (2021) produced a three-layered scaffold based on chitosan and alginate, which demonstrated excellent biocompatibility and the capacity to promote tissue regeneration.

#### 2.1.4 Dextran

**FIGURE 2:**
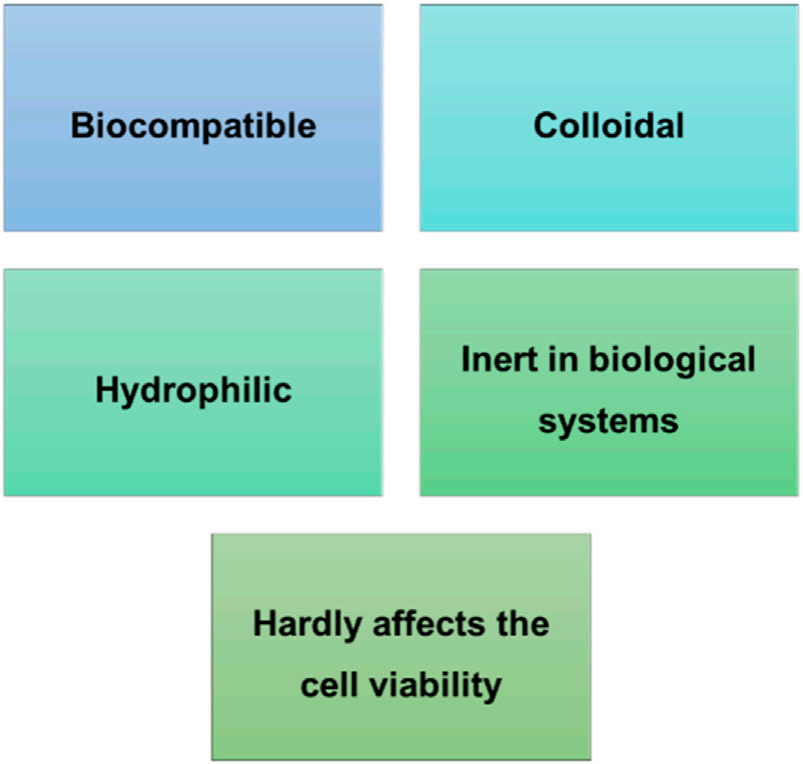
Dextran advantageous features.

Dextran is a hydrophilic natural polysaccharide with linear chains containing α-1,6 linked D-glucopyranose residues produced by several strains of bacteria. This polymer contains a large number of hydroxyl groups that can be easily conjugated to drugs and proteins by either direct attachment or through a linker, and its degradation occurs via phagocytosis (Suamte et al., 2023), (Mehvar, 2000). These polymer properties are described in Figure 2. Dextran-derived hydrogels can be prepared by chemical or physical cross-linking or even by radical polymerization (ter Horst et al., 2019). These hydrogels have restricted cell adhesivity and do not appear to influence cell viability, so they are normally used for drug delivery. In addition, dextran has a slow degradation rate, which limits its application for wound healing (ter Horst et al., 2019). Researchers have tried to alter dextran hydrogels properties to enhance cell adhesion (Massia and Stark, 2001) and improve biocompatibility (Sun et al., 2010). For that Möller et al. (2007) modified a dextran degradation profile by treating dextran with glycidyl methacrylate to improve its functionality as a tissue engineered scaffold for burn wound healing (Sun et al., 2011). Sun et al. (2011) developed an acellular dextran hydrogel and studied its application in full thickness burns using a mice model. This study revealed that the prepared hydrogel stimulates angiogenesis and skin regeneration. Shen et al. (2015) reported similar results in a full thickness porcine model, demonstrating an additional improvement in dermal reconstruction and reinnervation. Furthermore, Park et al. (2014) evidenced the capacity of a hypoxia-inducible dextran hydrogel to promote neovascularization. Regarding cell delivery to wounds, Voigt et al. (1999) seeded keratinocytes into collagen-coated dextran microspheres. These microspheres were transplanted as micrograft’s to full thickness wounds in an athymic nude mouse model, revealing the reconstitution of the epithelium, which was multi-layered and keratinized within 14 days. Yang et al. (2022) fabricated a multifunctional hydrogel created through polylysine-graft-cysteine/oxidized dextran for supporting skin tissue regeneration. This hydrogel exhibited not only good antibacterial and free radical scavenging capacity, but also a better efficacy for promoting wound repair than commercial dressing.

#### 2.1.5 Hyaluronic acid

**FIGURE 3:**
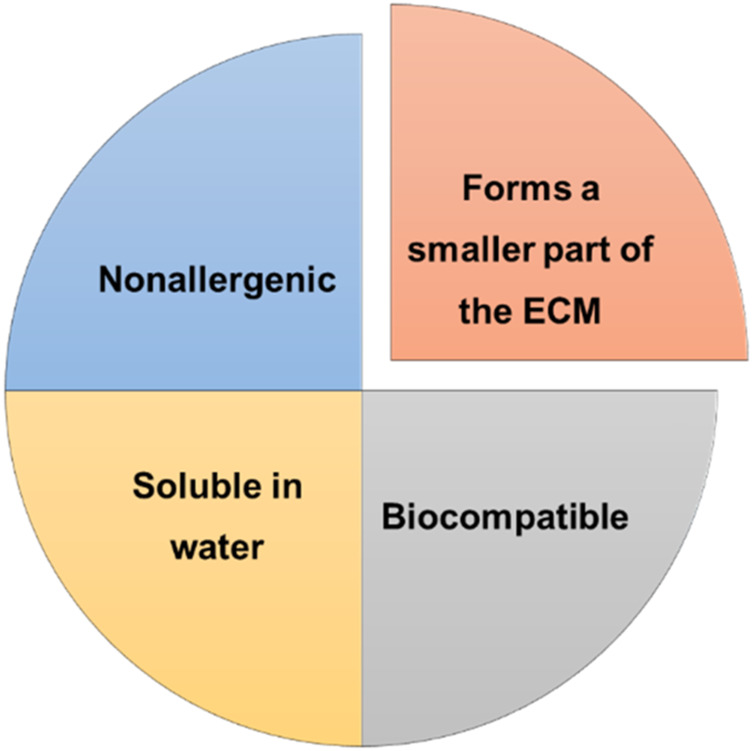
Beneficial properties of hyaluronic acid.

Hyaluronic acid is a non-sulfated glycosaminoglycan consisting of repeating polymeric disaccharides of D-glucuronic acid and N-acetyl-D-glucosamine linked by a glucuronidic β (1→3) bond (Weissmann and Meyer, 1954). It is an anionic, linear polymer that exists in all living organisms and can be found in connective tissue particularly in the dermis of the skin (ter Horst et al., 2019). This polymer has several beneficial properties that are described in Figure 3. Hyaluronic acid plays important roles in cell growth and differentiation, and it facilitates early inflammation, which is essential for wound healing (Suamte et al., 2023), (Chen et al., 2002). However, its main limitation is due to its rapid enzymatic degradation in physiological media (Negut et al., 2020). Due to its properties, easy preparation and modification, hydrophilicity, biodegradability and non-adhesiveness, this polymer is normally used for the development of cosmetic products, as well as, tissue scaffolds (Suamte et al., 2023), (Gravante et al., 2007). In addition, hyaluronic acid has physical and biochemical features either in solution or hydrogel form that improves body repair (Collins and Birkinshaw, 2013). Su *et al.* developed a decellularized scaffold of hyaluronic acid loaded with epidermal growth factor, which exhibited good healing rates (over 87%) on wounds with thick epidermis and skin appendage formation (Su et al., 2014). A study conducted by Sanad and co-workers demonstrated the potential of a novel chitosan–hyaluronic acid composite sponge scaffold enriched with andrographolide lipid nanocarriers for use in wound treatment. Indeed, it has been demonstrated by *in vivo* evaluation using a rat model that the developed scaffold heightened wound healing with no scar formation and improved tissue quality (Sanad and Abdel-Bar, 2017). Chang *et al.* developed an enzyme-crosslinked hyaluronic acid-tyramine hydrogel loaded with antioxidant and photothermal silver nanoparticles to be used as a functional wound dressing. This nanocomposite hydrogel has good antibacterial and antioxidant properties, decreases inflammation, supports angiogenesis, and accelerates the healing process. This study demonstrates the great potential of the developed hydrogel for the treatment of infected skin wounds (Chang et al., 2022).

#### 2.1.6 Starch

Starch is a storage polysaccharide that has interesting properties, such as biodegradability, non-cytotoxicity, and renewability (MORRISON et al., 1990). Starch is also easily modified by chemical procedures opening the door to its use for drug delivery and tissue engineering. Indeed, Ngoenkam et al. (2010) developed an injectable starch-chitosan hydrogel for the delivery of chondrocytes. Starch can be easily blended with other polymers due to its non-ionic nature. It can increase pore size and water uptake of the resulting polymer blend, as demonstrated in this study. Sundaram *et al.* also reported excellent mechanical properties with increased porosity (82.51%) of a gelatine-starch scaffold making it suitable for cell growth, vascularization and cellular interaction (Sundaram et al., 2008). Starch-based scaffolds have a good biodegradation rate and can act as a substrate for cell adhesion (Roslan et al., 2016). The potential for these scaffolds in wound healing applications has been highlighted by Waghmare et al. (2018) who showed an increase in mouse fibroblast cells (L929), growth and proliferation with low toxicity. Salehi et al. (2017) prepared starch-based nanocomposite hydrogel scaffolds with zeolite nanoparticles and chamomile extracts for the treatment of chronic ulcers. They demonstrated the successful healing of the refractory ulcers without any hypersensitivity reaction towards the scaffold. Chhabra et al. (2020) reported the development of starch-gelatine scaffolds for wound healing applications. This study revealed the ability of the scaffold to accelerate wound closure and promote tissue reorganization and remodelling.

#### 2.1.7 Agar

Agar is a polymer with high biocompatibility and non-immunogenicity (Zarrintaj et al., 2018), which can form a gel that mimics ECM physical characteristics of the tissue with the ability to transport cell growth factors to injured tissues and organs (Lewitus et al., 2011). Nayak and Gupta (2015) demonstrated that a binary blend of agar with keratin is non-toxic and allows cell growth, which makes it appropriate to be used in wound healing and skin regeneration. Furthermore, it has been confirmed that a novel squalene loaded agar-based emulgel scaffold can accelerate the healing of burn wounds by significantly contracting the wound area with high re-vascularization and macrophage polarization (Shanmugarajan et al., 2020). Eivazzadeh-Keihan et al. (2021) developed a nanobiocomposite scaffold based on cross-linked lignin-agarose hydrogel, extracted silk fibroin solution, and zinc chromite nanoparticles for wound healing applications. This study revealed that mice wounds treated with this nanobiocomposite were almost completely healed in 5 days. Basha et al. (2020) synthesized fumaric acid and incorporated it into an agar-silver hydrogel to tackle wound healing. This hydrogel showed a synergistic antibacterial effect against microbes, a quicker rate of wound healing, and augmented collagen deposition and angiogenesis.

#### 2.1.8 Silk

Silk is a natural fibrous protein acquired from the larvae of *Lepidoptera*, consisting of two key proteins: a fibrous protein and sericin which is soluble in water (Suamte et al., 2023), (Reddy et al., 2021). This biopolymer has several beneficial properties such as low weight, high strength, excellent elasticity and durability; and it can be used as a coating material or support for the development of biocompatible scaffolds (Choi et al., 2018). Wang et al. (2021) developed a bioactive scaffold composed of hyaluronic acid and natural silk fibroin nanofiber, which has a similar chemical and biophysical composition, as well as, a similar nanostructure to the ECM. This study revealed the cytocompatibility of the scaffold with cell proliferation and differentiation. The presence of this material increased wound healing with collagen assembly and inhibited scar formation. Babu et al. (2018) conducted a study involving the use of silk fibroin with silver oxide nanoparticles to evaluate their synergistic effect on wound healing. The developed biomaterial revealed a noteworthy antibacterial activity against pathogenic and non-pathogenic bacteria and demonstrated through an *in vitro* scratch assay that the T3T fibroblast cells quickly migrated to the scratch area with complete coverage of the area upon 24 h. This study also confirmed the biocompatibility of the scaffold through a cytotoxicity assay highlighting its potential for wound healing applications (Babu et al., 2018). Xie et al. (2022) developed an allantoin-functionalized silk fibroin-sodium alginate composite scaffold for the treatment of cutaneous wounds. The scaffolds revealed excellent biocompatibility and enabled the wound healing process by boosting collagen deposition, re-epithelialization, and vascularization at the wound site.

#### 2.1.9 Fibrin

Fibrin is an insoluble protein, formed by the cleavage of soluble fibrinogen by the proteolytic thrombin, which is activated upon tissue injury. Given the ability of fibrin to act as a blood clotting agent, it is natural that it forms a fibrous mesh (ter Horst et al., 2019). This polymer has been widely studied for wound healing applications (ter Horst et al., 2019). Fibrin-based scaffolds have demonstrated that they let binding of a series of active biological molecules to themselves which in turn enables specified cell matrix relations and augments tissue regeneration (Suamte et al., 2023). These scaffolds offer the required time for the development of neo matrix with a gradual reassimilation through proteases (Bencherif et al., 2017). Fibrin can also be used in combination with distinct polymers, as a biological scaffold, with potential biomaterial for the regeneration of primary cells, skin, and bones (Ahmed et al., 2008). The use of this polymer in the design of scaffolds, may not be ideal, as fibrin can be rapidly degraded (SAKIYAMA et al., 1999). Bastidas et al. (2020) prepared a fibrin-based scaffold composed of poly (lactic-co-glycolic acid) (PLGA) by electrospinning. They evaluated its potential to be used as a skin substitute, and results demonstrated that this scaffold presents blood compatibility and incorporation of fibrin improved cell adhesion and viability. Furthermore, the effect of an injectable fibrin scaffold loaded with growth factors for the repair of full thickness skin defects was assessed (Shao et al., 2021). Shao et al. (2021) showed that this scaffold, which has a fine network of platelets and white blood cells, allowed a sustained release of growth hormones, facilitating the cellular proliferation, revascularization and deposition of collagen. These results highlighted the potential of the scaffold to promote skin repair. Mirhaj et al. (2022) fabricated a polycaprolactone/keratin/platelet-rich fibrin fibrous scaffold by electrospinning and demonstrated its angiogenic potential, as well as its ability to accelerate wound healing, collagen deposition and formation of skin appendages.

#### 2.1.10 Collagen

**FIGURE 4:**
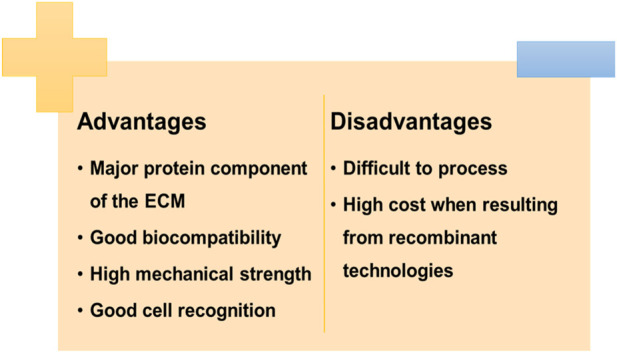
Advantages and disadvantages of using collagen.

Collagen is the most abundant protein in the human body and the major component of the extracellular matrix (Sherman et al., 2015). It is the main structural protein in the dermis, having an important role in wound healing. Thereby, collagen has been employed in the development of several skin scaffolds (Chattopadhyay and Raines, 2014). Collagen type I is the most used in skin scaffolds, since it is the most abundant protein in native dermal tissue (ter Horst et al., 2019). This polymer can easily be blended with other polymers such as chitosan, hyaluronic acid, and chondroitin sulfate to obtain improved biological and mechanical features (Matsiko et al., 2012). The properties of the scaffolds can be modulated by varying the concentration of collagen (Wozney et al., 1988). Collagen presents excellent properties, such as biocompatibility, low antigenicity, and high mechanical strength, and it facilitates cell binding, proliferation, differentiation, and production of ECM (Figure 4), which make it suitable for wound healing applications (Reis et al., 2008). Natarajan and co-workers developed tannic acid cross-linked collagen scaffold using a casting technique and evaluated its effects on wound healing (Natarajan et al., 2013). The study included an *in vivo* test using excision wound model rats, demonstrating improved healing rates, and an efficient wound closure. This new biomaterial also revealed the potential for controlled drug release. Indeed, Tezgel et al. (2020) loaded nanostructured lipid carriers and siRNA complexes in collagen scaffolds and coated them with a 1,2-Dioleoyl-sn-glycero-3-phosphoethanolamine (DOPE) shell. This study showed a prolonged release of siRNA with good transfection efficiency and a prolonged downregulation of ERK-1 protein for 7 days. These results confirmed that the developed collagen scaffolds are promising materials to be applied into wounds to promote healing through controlling the expression of proteins involved in this complex process (Tezgel et al., 2020). Kumar Reddy Sanapalli et al. (2022) developed an L-Glutamic acid loaded collagen/chitosan composite scaffold for the faster healing of diabetic wounds. The scaffolds have not exhibited any toxicity and showed higher rates of wound contraction, having also a modulatory effect on the inflammatory biomarker MMP-9.

#### 2.1.11 Fibronectin

Fibronectin is a high molecular weight glycoprotein obtained from human and bovine plasma that can be used as a scaffold to assist cell adherence and migration, thus regulating cell proliferation and differentiation (Suamte et al., 2023), (Pankov and Yamada, 2002). Chantre et al. (2018) produced scaffolds based on fibronectin nanofibers for wound healing applications. The obtained results revealed that this scaffold has the ability to accelerate wound closure, as well as, to increase tissue restoration and recovery of dermal and epidermal constructs including adipose tissue and skin appendages. Jara et al. (2020) synthesized a novel fibrin matrix composed of fibronectin and plasma fibrinogen combined with fibrin nanofibers with fibronectin nanoclusters. These nanoclusters stimulated the pro-healing cells such as endothelial cells, fibroblasts and keratinocytes and promoted their migration, survival, proliferation and function, resulting in quicker and improved wound healing.

#### 2.1.12 Elastin

Elastin is another ECM protein that is hydrophobic and insoluble in nature. This polymer can be used in the development of biomaterials in its different forms: hydrolyzed soluble elastin, insoluble elastin fibres, recombinant tropoelastin, block copolymers of elastin, repeats of synthetic peptide sequences and in combination with other polymers (Suamte et al., 2023), (Daamen et al., 2007). Boekema et al. (2014) developed a novel collagen-elastin scaffold to be used as a dermal replacement for treating full-thickness skin defects such as burn wounds. Furthermore, a study reported that a triple-polymer scaffold made of a collagen-elastin-polycaprolactone composite was developed (Chong et al., 2019). The inclusion of elastin was revealed to decrease the stiffness of the scaffold, while also decreasing hysteresis and increasing elasticity. Additionally, the developed scaffold promoted keratinocyte and fibroblast proliferation, tissue integration and accelerated early-stage angiogenesis. Thus, this study revealed the potential use of the triple-polymer scaffold in supporting full growth with tissue regeneration in a severe burn injury (Chong et al., 2019).

#### 2.1.13 Keratin

Keratin is a fibrous protein present in skin, nails and hair. It has interesting properties, like good biocompatibility and the ability to form self-organized structures that synchronize cellular recognition and behavior (Bragulla and Homberger, 2009), (Rouse and Van Dyke, 2010). Moreover, wool keratin has shown its ability to promote cell adhesion, due to the presence of the cell adhesion sequences, arginine-glycine-aspartic acid and leucine-aspartic acid-valine (Tachibana et al., 2002). Xu et al. (2013) prepared a human hair keratin scaffold by freeze-dying and demonstrated its ability for subcutaneous implantation and for treating full-thickness skin defects in rats. Indeed, this study confirmed the potential of keratin for skin regeneration since the scaffold provided a more rapid vascularization, thicker epidermis, lower contractions and formation of new hair follicles. A study involving the preparation of a porous keratin/chitosan scaffold by lyophilization revealed enhanced antibacterial activity and cell proliferation rates, highlighting its promising use for wound dressings (TAN et al., 2015). The development of an *in situ* forming keratin hydrogel and its application as a drug depot for wound repair revealed that the deferoxamine-loaded scaffold accelerated healing in the full-thickness wounds of streptozotocin-induced diabetic rats by raising angiogenesis and neovascularization in wounds (Chen et al., 2021).

#### 2.1.14 Gelatine

Gelatine is a natural polymer obtained from collagen and it has beneficial features such as low antigenicity, biocompatibility and biodegradability making it suitable for medicinal and pharmaceutical applications (Young et al., 2005). This polymer has acidic and basic groups giving it a high flexibility in terms of its structure in comparison to other polymers. Gelatine can be processed in several forms, for example as injectable hydrogels, sponges and microspheres, given its gelation abilities and ease of processibility (Malafaya et al., 2007). Moreover, other peptides and cell adhesion molecules can be incorporated into gelatine for its use in distinct tissue engineering approaches (Ito et al., 2003). Ninan and co-workers developed porous gelatine/zeolite scaffolds by freeze drying, revealing their cytocompatibility and antibacterial activity, as well as, their ability to improve wound healing in Sprague Dawley rats (Ninan et al., 2014). A study described the development of a gelatine-based scaffold combined with alginate hollow fibers, to attain a vascular network for nutrients and oxygen supply for embedded cells. In this study, human umbilical vein endothelial cells were embedded into the scaffolds and the network formed by the hollow fibers supplied the oxygen and required nutrients and aided the waste removal (Li et al., 2020). Sadeghi et al. (2023) developed a gelatine/alginate sulfate hybrid scaffold as a dermal substitute to hasten the healing of full-thickness diabetic ulcers in a diabetic mouse model. The scaffolds revealed no cytotoxicity, as well as, an increase in cell growth. This increase was related to the increase in the alginate sulfate content. Additionally, this study confirmed the potential of hybrid scaffolds to act as dermal substitutes, as they lead to the formation of an epidermal layer with a homogeneous distribution of collagenous tissue and low penetration of immune cells.

### 2.2 Synthetic polymers

In contrast to natural polymers, synthetic polymers are chemically synthesized. Therefore, its properties can be adjusted in a controlled manner, having constant and homogeneous physico-chemical properties and stability. These polymers do not enclose impurities, being usually mechanically stable and with controlled degradation (Negut et al., 2020). They have several biological properties, namely, biocompatibility, chemical structure versatility, tuneable mechanical properties, and degradation rate. Moreover, some synthetic polymers (e.g., polyesters) can be biodegradable, and, in general, these materials are cost-effective compared to natural polymer preparation (Kohli et al., 2019). However, synthetic polymers also have limitations like an associated toxicity risk, and they do not offer a therapeutical advantage like natural polymers, as they are biologically inert (Negut et al., 2020), (Zhong et al., 2010). In addition, these materials have relatively poor cellular interaction, so they normally require surface treatment or are blended into a composite material (e.g., natural polymers) to enhance their cellular compatibility (Kohli et al., 2019). These surface treatments generally included chemical alterations to reduce hydrophobicity, like inserting polar groups on the polymeric surface increasing direct cell adhesion and/or the addition of a biological component like adhesion peptides (Kohli et al., 2019). The most commonly used strategy consists of the combination of synthetic and natural polymers, as natural polymers contribute with their natural biological activity and their proximity to the ECM tissues (Davison-Kotler et al., 2018; Sharma et al., 2017; V Shevchenko et al., 2010).

#### 2.2.1 Polydimethylsiloxane

**FIGURE 5:**
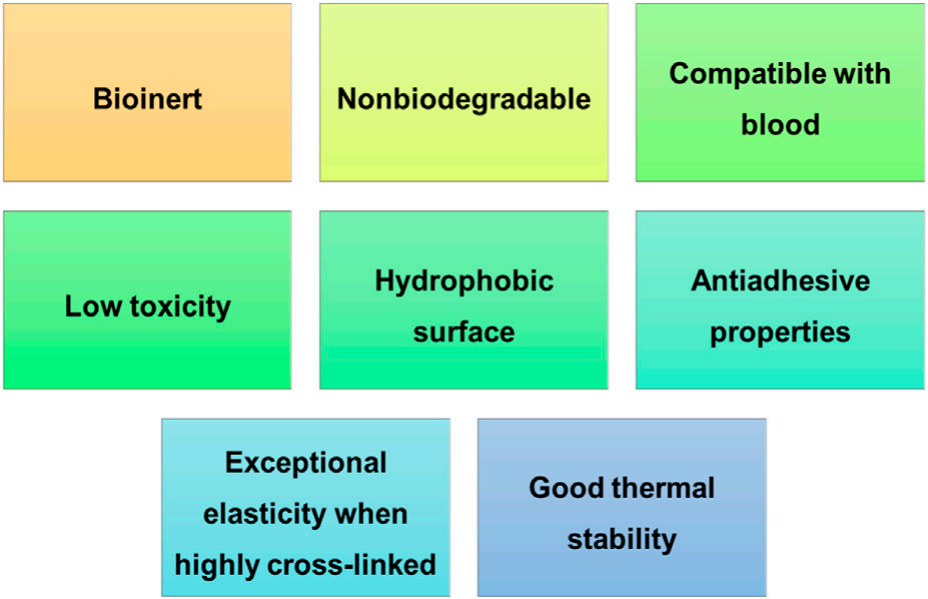
Polydimethylsiloxane properties.

Polydimethylsiloxane (PDMS) is a part of the siloxane (silicone) family and is the most commonly silicone elastomer used (Kohli et al., 2019). This polymer has several properties, including its inertia, compatibility with blood, low toxicity, low glass transition temperature, among others (Figure 5) (Akhtar et al., 2016), (Polmanteer, 1988). Given its beneficial features, PDMS is used in several applications, like in cardiac pacemaker leads, artificial skin, contact lenses, medical adhesives, finger joints, catheters and drug delivery systems (Akhtar et al., 2016)– (Leeper and Wright, 1983). Regarding the tissue engineering field, Nikpour and co-workers developed a curcumin-loaded Fe(II) metal–organic framework/polydimethylsiloxane sponge that showed its ability to enhance healing and revascularization, highlighting its potential to be used as a 3D porous substrate for tissue engineering and regenerative medicine (Nikpour et al., 2022).

#### 2.2.2 Polyvinyl Alcohol

Polyvinyl Alcohol (PVA) is a water-soluble polymer with a repeating hydroxyl group in its backbone (Kohli et al., 2019). This polymer has desirable features such as biodegradability, biocompatibility, and non-toxicity which make them suitable to be used in several biomedical applications (Allen et al., 2004). PVA is frequently used in conjugation with other polymers, such as chitosan and polyhydroxy butyrate, for the production of nanofibers that can be used in tissue engineering applications (e.g., wound healing) (Rahmani Del Bakhshayesh et al., 2018). Ahlawat et al. (2019) developed *Carica papaya*-loaded PVA/Gelatine nanofibers for wound dressing application. This study revealed that the fabricated material provided a moist environment at the wound site, allowing re-epithelialization. In addition, the cytotoxicity assay performed on NIH 3T3 fibroblast cells demonstrated that the nanofibers have good compatibility with no cytotoxic effects. It is also worth noting the excellent antibacterial activity of the material against gram-positive and gram-negative bacteria, evidencing the promising use of these nanofibers in wound healing applications. Rajora and colleagues prepared a nanofiber mat of neem gum polysaccharide with PVA using an electrospinning technique (Rajora and Bal, 2022). This nanofiber showed hemocompatibility, as well as, biodegradability having antimicrobial activity against *Escherichia coli* and *Staphylococcus aureus*. Furthermore, this study revealed that the prepared device speed up wound healing in mice with dense collagen and fibroblasts highlighting.

#### 2.2.3 Poly-N-Vinylpyrrolidone

Poly-N-vinylpyrrolidone (PVP) is a biocompatible, water-soluble, and biodegradable polymer extensively used as a hydrogel membrane for skin alternative products, since it is no irritating to the skin (Kamoun et al., 2017). PVP also has other beneficial features, such as its environmental stability, low cytotoxicity, high chemical and thermal resistance, affinity to complex hydrophilic and hydrophobic substances and very good solubility in organic solvents (Negut et al., 2020). Moreover, the treatment of PVP through coating methods and copolymers combination gives it a variety of features (e.g., prevention of microbial penetration) useful for wound healing applications (Kohli et al., 2019). A study reported the development of a phlorizin-loaded silk protein/PVP composite nanofibrous membrane to be used as a wound dressing. The study demonstrated its ability to promote collagen deposition and angiogenesis in the wound site, as well as, its capacity to increase autophagy, accelerating wound repair (Sun et al., 2022). Zhang and co-workers prepared a chitosan, PVP, and dihydroquercetin nanofiber film to be used as wound excipients. The fibre film showed antioxidant and antibacterial activity, acceleration of the healing process and promotion of wound healing by inducing the autophagy pathway. The cytotoxicity assay performed on keratinocytes (HaCat cells) proved that the film is non-toxic (Zhang et al., 2022).

#### 2.2.4 Polyurethane

Polyurethane (PU) has versatile features such as toughness, durability, biocompatibility, and degradation rates which can be customized depending on the application (Zhang et al., 2016). PU can be employed in several combinations such as with olive oil to obtain antioxidant properties and a photoprotective mechanism or combined with propolis to enhance antibacterial activity and mechanical strength (Amna et al., 2014), (Kim et al., 2014). The combination of PU with dextran fibres through electrospinning revealed good angiogenesis activity alongside high anti-inflammatory action leading to the acceleration of cutaneous wound healing (Unnithan et al., 2015). A study, in which a nanocomposite PU/polycaprolactone scaffold with graphene oxide was developed through electrospinning, revealed great biocompatibility in contact with skin fibroblast cells and improved hydrophilicity (Sadeghianmaryan et al., 2020). Jaganathan *et al.* fabricated an electrospun PU scaffold loaded with nanofibers of zinc nitrate and evaluated its use as a wound dressing material (Jaganathan and Mani, 2019). The cytocompatibility and hemolytic study demonstrated a lower hemolytic index with increased fibroblasts proliferation. The developed material also exhibited enhanced physiochemical properties. Therefore, this material seems to be a good alternative for wound dressing materials. A PU cellulose acetate electrospun fibbers loaded with rosemary essential oil and adsorbed silver nanoparticles exhibited antibacterial activity and improved hydrophilicity of the fibbers, leading to a better attachment of cells to the micro-nanofibers, similar to the natural ECM (Rather et al., 2023).

#### 2.2.5 Polylactic acid

Polylactic acid (PLA) is a biodegradable polyester obtained from renewable sources, like corn, starch, or sugar cane (Kohli et al., 2019), (Avinc and Khoddami, 2009). This polymer has several characteristics: inexpensive, non-toxic, hydrophobic, structurally stable, and excellent biocompatibility and mechanical integrity making it suitable for medical applications. PLA is an aliphatic polyester of naturally occurring lactic acid, thus, its degradation by enzymes or hydrolysis under physiological conditions originate products that are easily absorbed by the body (Avinc and Khoddami, 2009; Ulery et al., 2011; Sin et al., 2013; Yoon et al., 2017). Regarding tissue engineering applications, a novel dressing scaffold composed of Polyvinylpyrrolidone/PLA-Polyethylene oxide fibers with encapsulated collagen and cefazolin was evaluated as a wound healing material. Different scaffolds were prepared by varying the collagen concentration (10%, 20%, and 40% w/w based on PVP). The study showed that the scaffold adhesion rises with collagen increase boosting the healing effect. Moreover, the fabricated material exhibited antimicrobial activity and *in vivo* histological tests revealed that 10% and 20% collagen doses had the highest healing rate (Hajikhani et al., 2021). Another study reported the encapsulation of hydroxyapatite, zirconia, and graphene oxide nanosheets in mono, di, or tri phases, into nanofibrous scaffolds of PLA. It has been demonstrated that the scaffolds exhibited enhanced antibacterial activity and promoted cell viability. In addition, human fibroblasts proliferate on its surface, as well as, in the pores of the nanofibrous scaffold (Al-Wafi et al., 2021).

#### 2.2.6 Polyglycolic acid

Polyglycolic acid (PGA) is a biodegradable polymer that belongs to the family of linear aliphatic polyesters (Kohli et al., 2019). This polymer is biocompatible, more hydrophilic than PLA and has high tensile strength (Negut et al., 2020). However, the use of PGA in biomedical applications is limited by its rapid degradation through hydrolysis, which results in a pH decrease in its microenvironment due to carbon dioxide production, leading to local cell and tissue necrosis. Additionally, the excess glycolic acid production from PGA degradation can elicit an inflammatory response (Ceonzo et al., 2006), (Pihlajamäki et al., 2006). Sekiya and collaborators reported that fusing PGA with collagen decreased inflammation and induced neovascularization in a mouse skin defect model (Sekiya et al., 2013). The incorporation of sodium tripolyphosphate into PGA scaffolds fillers via CO_2_ foaming relieved inflammation and enhanced phagocytosis in a porcine model (Zhang et al., 2020).

#### 2.2.7 Poly (lactic-co-glycolic) acid

**FIGURE 6:**
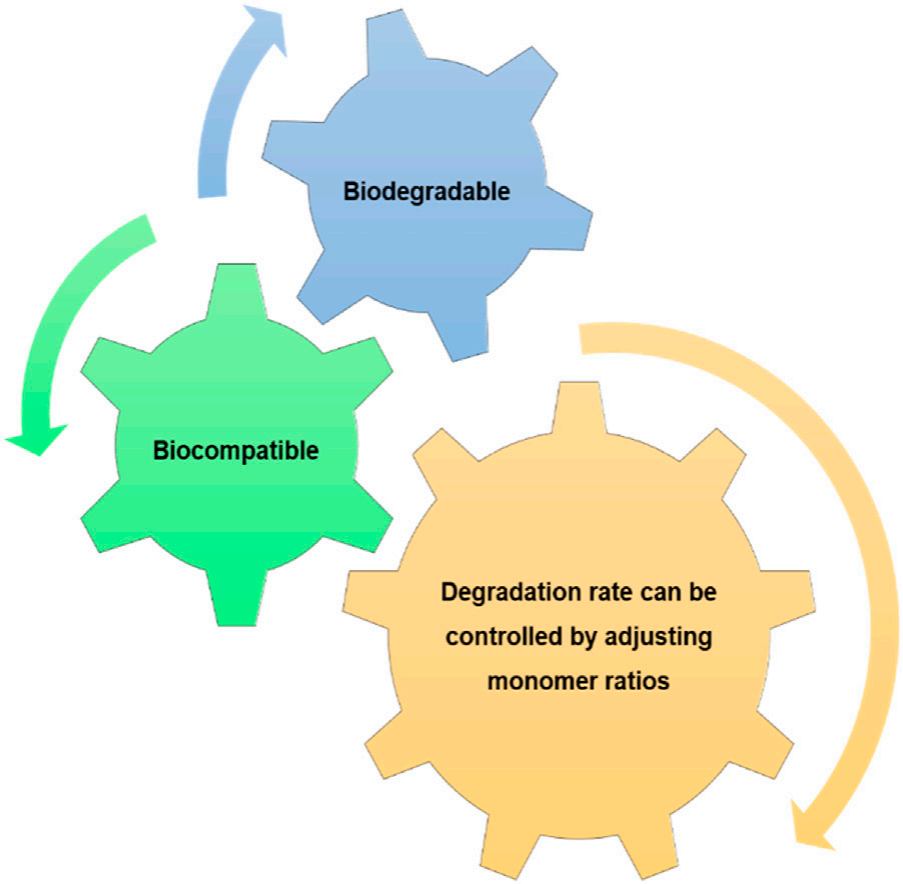
Poly(lactic-co-glycolic) acid properties.

Poly(lactic-co-glycolic) acid (PLGA) is a biodegradable polyester copolymer manufactured from random copolymerization of PLA and PGA (Kohli et al., 2019). The main properties of this polymer are described in Figure 6 (Negut et al., 2020). Given its beneficial features, it has been extensively used for sutures, drug delivery devices, and tissue engineering scaffolds (Maitz, 2015), (Pan and Ding, 2012). The preparation of a PLGA-liposome scaffold loaded with microRNA 145 and platelet-derived growth factor promoted angiogenesis *in vitro* and wound healing *in vivo* (Hu et al., 2021). Silk sericin-PLGA scaffolds obtained by electrospinning with ketoprofen for the treatment of periodontal diseases were developed by Chachlioutaki et al. (2022). The scaffold had enhanced hydrophilicity and mechanical strength, as well as, an anti-inflammatory effect on LPS-stimulated RAW-264.7 cells. Additionally, the developed material favours attachment and proliferation of human gingival fibroblasts (Chachlioutaki et al., 2022).

#### 2.2.8 Poly-ε-caprolactone

Poly-ε-caprolactone (PCL) is an extremely tough aliphatic polymer with good solubility, biocompatibility, exceptional elasticity and mechanical characteristics, low melting point and low decomposition rates along with non-toxicity and cost-effectiveness (Suamte et al., 2023), (Woodruff and Hutmacher, 2010). However, the main limitation of PCL is its low bioactivity and high hydrophobicity leading to the decrease of cell affinity with minimal tissue regeneration. Aiming to minimize these limitations PCL can be combined with other polymers like PLA to form a lesser hydrophobic construct with better degradability and mechanical properties (Patrício et al., 2013). For tissue engineering applications, a bilayer nanofibrous scaffold consisting of fish collagen and PCL produced using electrospinning, with the covalent attachment of chitooligosaccharides via carbodiimide chemistry demonstrated *in vitro* and *in vivo* assays and enhanced healing process (Chandika et al., 2021). Ekambaram et al. (2022) developed a novel wheatgrass extract infused PCL/chitosan nanofibrous scaffold through electrospinning for wound infections treatment and enhanced wound healing. The material revealed cytocompatibility against vero cells and exhibited higher anti-bacterial activity than commercial anti-biotic gentamicin. Additionally, the scaffold exhibited higher affinity with binding energy of−9.6 kcal/mol towards wound the healing receptor COX-2. This fact is very important as COX-2 induction enhances faster wound healing.

#### 2.2.9 Polyethylene Glycol

Polyethylene Glycol (PEG) is a biocompatible, nonbiodegradable, bioinert and hydrophilic polymer that has optimal biological and physicochemical properties, as well as, resistance to protein adsorption (Kohli et al., 2019), (Negut et al., 2020). Given the easiness to control its architecture along with its chemical composition, this polymer is an attractive scaffold material for tissue engineering applications (Suamte et al., 2023). A study in which an exosome secreted by adipose-derived stem cell was loaded into the matrix of a metalloproteinase degradable PEG smart hydrogel promoted diabetic wound healing by improving cellular functions (Jiang et al., 2022). Jin et al. (2021) developed a hybrid hydrogel composed of chitosan and poly (D,l-lactide)-PEG-poly (D,l-lactide) that tightly adhered to the skin with good antibacterial properties and accelerated the wound healing process.

## 3 Currently available biomaterials for skin regeneration

Tissue engineered skin substitutes have emerged as excellent alternatives to traditional wound healing strategies and tissue regeneration. It should be noted that skin was the first engineered organ that went from the bench to patient care (Rheinwald, 1989). Over the last few years, several synthetic and bioengineered substitutes have been prepared. Usually, these substitutes are placed within the wound providing several benefits, like the provision of barrier function along with protection against microorganisms, pain reduction and wound healing promotion (Alonso and Fuchs, 2003)–(de Mel et al., 2012). The research from the past years led to the commercialization of several skin substitutes, which can be classified in different ways (V Shevchenko et al., 2010), (Clark et al., 2007), (Horch et al., 2005; Atiyeh and Costagliola, 2007; MacNeil, 2007). They can be classified according to their cover duration as permanent, semi-permanent, or temporary, according to their anatomical structure as epidermal, dermal, or dermo-epidermal (composite), based on their composition as cellular or acellular, and according to the type of biomaterial used as biological (autologous, allogeneic, xenogeneic) or synthetic (biodegradable, non-biodegradable). Synthetic skin substitutes are composed of acellular materials, usually used as a barrier to fluid loss and microbial contamination. These skin substitutes normally comprise a nylon mesh or collagen that acts as a “dermis” and a silicon membrane that acts as an “epidermis” (Vig et al., 2017). They are used principally as temporary skin substitutes for superficial or mid-dermal partial thickness wounds and burns (Shakespeare and Shakespeare, 2002), (Shakespeare, 2005). Biobrane^®^, Integra^®^ and Alloderm^®^ are some of the synthetic acellular skin substitutes frequently used. On the other hand, the natural skin substitutes are primarily cultured allogeneic or autologous cell suspensions or sheets, which can be used on their own or along with a dermal matrix (Vig et al., 2017). Cellular allogeneic skin substitutes are generally developed using living neonatal foreskin fibroblasts with a mesh or matrix. These biomaterials have been used for the treatment of venous and diabetic ulcers, as well as, for wound management in epidermolysis bullosa, skin cancer, and in burns (Horch et al., 2005), (Supp and Boyce, 2005). Dermagraft^®^, Apligraf^®^, TransCyte^®^ and OrCel^®^ are some examples of commonly used natural skin substitutes with allogeneic cells. In the case of cellular autologous skin substitutes, there are two types available - Cultured Epidermal Autograft (CEA) and Cultured Skin Substitutes (CSS) (Vig et al., 2017). CEA involves the culture of autologous keratinocytes, derived from the skin biopsy of the patient (Supp and Boyce, 2005), while CSS is an autologous graft with both epidermal and dermal components. Epicel^®^ is an example of a skin substitute with autologous cells. Table 2 lists some of the commercially available skin substitutes, including those already mentioned.

**TABLE 2:** Commercially available skin substitutes.

Substitute type	Product	Components	References
Acellular	Alloderm^®^	Human acellular lyophilized dermis	Horch et al. (2005), Bello et al. (2001); Supp and Boyce. (2005); Gordley et al. (2009); Nathoo et al. (2014)
	Biobrane^®^	Ultrathin silicone as epidermal analog film and 3D nylon filament as dermal analog with type I collagen peptides	V Shevchenko et al. (2010), Horch et al. (2005), Supp and Boyce. (2005)
	SureDerm	Human acellular lyophilized dermis	Vig et al. (2017)
	Integra^®^ DRT (dermal regeneration template)	Dermal analog—bovine collagen and chondroitin-6-sulfate GAG; epidermal analog—silicone polymer polysiloxane	V Shevchenko et al. (2010), Horch et al. (2005), Supp and Boyce. (2005), Bello et al. (2001)
	OASIS Wound Matrix	Porcine acellular lyophilized small intestine submucosa	Hodde et al. (2005)
	AlloPatch^®^	Human allograft skin minimally processed to remove epidermal and dermal cells	Snyder et al. (2020)
	AlloSkin™ RT	Meshed human dermal graft is a sterile skin graft with broad clinical applications for acute and chronic wound therapy	Snyder et al. (2020)
	Epifix^®^	Dehydrated human amnion/chorion membrane (dHACM) allograft. EpiFix is a bioactive tissue matrix allograft composed of dHACM that preserves and contains multiple ECM proteins, growth factors, cytokines, and other specialty proteins	Snyder et al. (2020)
	NuShield^®^	Dehydrated placental allograft designed to protect and support healing in a variety of wound sizes and types	Snyder et al. (2020)
	Helicoll™	Acellular bovine collagen matrix free of contaminants	Snyder et al. (2020)
	Talymed^®^	Talymed advanced matrix is composed of shortened fibers of poly-N-acetyl glucosamine isolated from microalgae	Snyder et al. (2020)
	Hyalomatrix^®^ tissue reconstruction matrix	Nonwoven pad composed of a wound contact layer made of a derivative of hyaluronic acid in fibrous form with an outer layer composed of a semipermeable silicone membrane. The wound contact layer is biodegradable, and it acts as a 3D scaffold for cellular invasion and capillary growth. The silicone layer controls water vapor loss and provides protective coverage of the wound	Snyder et al. (2020)
	AltiPly^®^	Lyophilized Placental Membrane. The growth factor-rich matrix, with an outer basement membrane and epithelial layer, immediately serves as a scaffold for reepithelialization	Snyder et al. (2020)
Epidermal			
Autologous	Epicel^®^	Sheets of autologous keratinocytes attached to petrolatum gauze support	Carsin et al. (2000)
	MySkin	Cultured keratinocytes (subconfluent cell sheet) silicone support layer with a specially formulated surface coating	Haddow et al. (2003)
	EpiDex	Cultured keratinocytes from the outer root sheath of scalp hair follicles (confluent cell sheet)	Tausche et al. (2003)
	CellSpray	Non-/cultured keratinocytes (subconfluent cell suspension)	Magnusson et al. (2007), Goedkoop et al. (2010), Kirsner et al. (2012)
	Laserskin^®^ or Vivoderm	Autologous keratinocytes and fibroblasts, grown on microperforated hyaluronic acid membranes	Caravaggi et al. (2003)
Dermal			
Autologous	denovoDerm™	Autologous dermal substitute	Varkey et al. (2015)
	Hyalomatrix PA	HYAFF (an ester of hyaluronic acid) layered on a silicone membrane	Vig et al. (2017)
	Hyalograft 3D	Cultured fibroblasts hyaluronic acid membrane	Límová (2010), Uccioli (2003)
Allogeneic	Dermagraft^®^	Bioabsorbable polygalactin mesh matrix seeded with human neonatal fibroblasts and cryopreserved	Varkey et al. (2015)
	TransCyte^®^	Collagen-coated nylon mesh seeded with allogeneic neonatal human foreskin fibroblasts	Noordenbos et al. (1999)
	Hyaluronan-FNfds hydrogel matrix	Hyaluronan coupled with fibronectin functional domains	Myers et al. (2007)
	Terudermis	Silicone, bovine lyophilized crosslinked collagen sponge made of heat-denatured collagen	Groeber et al. (2011)
	Cyzact (ICX-PRO)	Cultured allogeneic human dermal fibroblasts embedded in a human fibrin gel matrix	Vig et al. (2017)
	Tegaderm-nanofibre Construct	Cultured dermal fibroblasts poly(e-caprolactone)/gelatin nanofibrous scaffold electrospun on polyurethane dressing	Vig et al. (2017)
	Human hair keratincollagen sponge	Cryomilled porcine acellular diisocyanate cross-linked dermis	Vig et al. (2017)
	Grafix^®^	Cryopreserved amnion or chorion matrix retaining the extracellular matrix, growth factors, and endogenous neonatal mesenchymal stem cells, fibroblasts and epithelial cells of the native tissue	Snyder et al. (2020)
Xenogeneic	Matriderm	Bovine non-cross-linked lyophilized dermis, coated with a-elastin hydrolysate	Límová (2010), Cullen et al. (2002)
	Collatamp	Multilayer bovine collagen matrix	Vig et al. (2017)
	Permacol Surgical Implant	Porcine acellular diisocyanate crosslinked dermis	Límová. (2010), Cullen et al. (2002)
	Bovine collagen cross-linked with microbial transglutaminase	Freeze-dried bovine collagen scaffold cross-linked with microbial transglutaminase	Still et al. (2003), Stark et al. (2006), Ackermann et al. (2010)
	EZ DermTM	Porcine aldehyde cross-linked reconstituted dermal collagen	Bello et al. (2001)
Synthetic	Hybrid nanofibrous PLGA/chitosan Membrane	PLGA/chitosan hybrid electrospun nanofibrous membrane	Ng and Hutmacher. (2006), Lindberg and Badylak. (2001)
	Biodegradable polyurethane microfibers	Biodegradable polyurethane microfibres	Rockwood et al. (2007)
Epidermal/Dermal (Composite)			
Autologous	Tiscover™ (A-Skin)	Autologous full thickness cultured skin	Varkey et al. (2015)
	PolyActive	Cultured keratinocytes and fibroblasts polyethylene oxide terephthalate (PEO)/polybutylene terephthalate (PBT)	van Dorp et al. (1999)
	Permaderm™ (Cincinnati Shriners Skin Substitute)	Autologous fibroblasts and keratinocytes in culture with bovine collagen and GAG substrates	Varkey et al. (2015)
	denovoSkin™	Autologous full thickness substitute consisting of dermal and epidermal layers	Varkey et al. (2015)
	TissueTech Autograft System (Laserskin and Hyalograft 3D)	Cultured keratinocytes and fibroblasts microperforated hyaluronic acid membrane	(Caravaggi et al., 2003), (Límová, 2010)
Allogeneic	Apligraf^®^	Bovine collagen matrix seeded with neonatal foreskin fibroblasts and keratinocytes	Hellman (2006), Halim et al. (2010), Ferreira et al. (2011), Dodson and Levine (2015)
	OrCel^®^	Type I collagen matrix seeded with neonatal foreskin fibroblasts and keratinocytes	Still et al. (2003), Stark et al. (2006), Ackermann et al. (2010)
	StrataGraft™	Full thickness skin substitute with dermal and fully differentiated epidermal layers	(Límová, 2010), (Supp et al., 2004; Schurr et al., 2009; Centanni et al., 2011)
	Celaderm™	Sheets of cells derived from neonatal (allogeneic) foreskin	Hellman (2006), Halim et al. (2010), Ferreira et al. (2011), Dodson and Levine (2015)
	Karoskin (Karocells)	Native human cadaver skin with dermal and epidermal cells	V Shevchenko et al. (2010)
Xenogeneic	Oasis^®^	Intact matrix from porcine small-intestine submucosa and intended for wound closure stimulation in acute, chronic and burns wounds	Límová (2010), Cullen et al. (2002)

## 4 New directions for the advance of skin regeneration field

Being one of the most complex processes in the human body, wound healing involves spatial and temporal synchronization of the inflammatory phase, with tissue regeneration and remodeling phases (Gethin, 2012), (Velnar et al., 2009).

Nowadays, skin wound treatment follows a combination between regeneration strategies and conventional treatments, like debridement (Tottoli et al., 2020). Wound treatment is still a challenging field, for example the difficulty to use skin grafts for the replacement of surface deficits, due to the impossibility to fixed them by simply approaching it to the injured skin margins. Also, skin grafts are major surgical procedures that are Invasive and can expose the patients to severe complications, since there will be an area of the skin that will know be exposed due to the skin that was removed to cover the wound (Dunkin et al., 2007). Regenerative skin wound healing is a novel and fast developing medical science in the biomedical field that seeks to restore skin to its original function, aiming to reestablish injured cells and skin. It focuses on the enhancement of the regeneration process using different strategies, based on a reparative approach to the physiological process of wound healing, minimizing the scarring (Boyce and Lalley, 2018), (Pang et al., 2017).

There are key components in regenerative medicine, particularly for wound healing, namely growth factors, involved in the stimulation of the wounds (WERNER and GROSE, 2003); cellular skin substitutes, that provides all the elements needed for skin regeneration (e.g., cells, mediators and materials that mimic the extracellular matrix (ECM)) (Kallis et al., 2018); gene therapy, for the prevention or treatment of skin diseases (for example, the use of holoclones in a patient with junctional epidermolysis, a disease that results in fatal skin adhesion disorder) (Mavilio et al., 2006; Aragona and Blanpain, 2017; High and Roncarolo, 2019); induced pluripotent stem cells (iPSCs), where the cell reprograming technology consists in reprogramming the adult stomatic cells into those iPSCs. iPSCs are expected to have a major role in regenerative medicine as an important source for transplant therapy, since *in vitro* and *in vivo* studies on mouse models have demonstrated the vast potential offered by them. iPSCs can generate a number of human autologous cells for degenerative skin disorders and advanced chronic wound treatment (Takahashi et al., 2007; Hu et al., 2010; Villa-Diaz et al., 2012; Zhang et al., 2015; Wang et al., 2019); skin tissue engineering, consisting in different methods to promote biological regeneration, repair the tissues, or replace tissues functions with systems that contain living tissue or cells. The variety of elements in the tissue engineering area comprises biomaterials, growth factors, cells, and engineering components such as scaffolds, tubes, bioreactors, oxygenators and pumps (Ehrenreich and Ruszczak, 2006).

Scaffolds are the best strategy to restore, maintain and improve tissue functionality in wound treatments according to the definition of tissue engineering as described by the National Science Foundation seminar. Scaffolds have a specific role in the reparation and regeneration of tissues, providing a foundation for cell survival, proliferation and differentiation (O’Brien, 2011), (Chaudhari et al., 2016). Scaffolds should be as similar as possible to the native ECM since all cells are constantly in contact with the ECM. Isolated cells are not capable of spontaneously organizing themselves into new tissues. Furthermore, the ECM is widely known to offer structural support to cells and tissues (Hynes, 2009). Scaffolds can be produced using natural and/or synthetic biomaterials. Materials which can remain stable in a biological environment and/or from materials that can be degraded in the human body (Chaudhari et al., 2016), (Chen and Wang, 2002). The use of synthetic materials has several advantages: production on large scales, the ability to control the material’s molecular weight and degradation time, adapting its properties to different applications; their tunable chemical properties, by cross-linking for example; and their biocompatibility and biodegradability properties (Eltom et al., 2019). Polymers are the most frequently used material in tissue engineering, particularly biodegradable polyesters, which have greater compatibility with body tissues. Synthetic polymers such as PLA, PGA, PLGA and PCL can form matrices individually or as composites. These polymers have been used due to their process easiness, adjustable mechanical and chemical properties, and their low cost. A work conducted by Ferri et al. (2018) analyzed cell growth and healing effectiveness in composite systems containing synthetic polymers and bioactive substances, where bioactive glasses were mixed with PLGA copolymer and the results showed encouraging evidence *in vitro* and *in vivo* scaffolds neovascularization. In order to overcome the previously described limitations of synthetic materials, natural materials have been studied since they have good biocompatibility and biodegradability when compared with synthetic materials. Some natural materials contain signal sequences that promote and maintain cell attachment, function, and structure. Natural polymers such as silk, fibrin, collagen, and gelatin are the most used natural biomaterials, particularly for protein derivation and are intended for tissue engineering, because these polymers are already present in the human body as ECM elements (Tottoli et al., 2020). Recently, a collagen-based scaffold developed in the form of a wound graft, marketed as Integra, was approved by the U.S. Food and Drug Administration for the treatment of severe burns. The new treatment decreased patients’ hospital stays (Iovene et al., 2021), (Ryan et al., 2002).

Recently natural polymers have been studied in combination with synthetic polymers, or even combined with drugs and bioactive molecules. The combination of these materials improves bioactivity, including mechanical and chemical properties, and the chemical release is more controlled for regenerative medicine. Singaravelu *et al.* conducted a study where a complex matrix made by a porous keratin-fibrin-gelatin 3D sponge scaffold was designed and the drug mupirocin was incorporated for tissue engineering applications (Frykberg and Banks, 2015). For tissue regeneration the combination of multi-polymer-type materials in their composition offers the capability to adjust mechanical strength, degradation rate, chemical properties, and cellular adhesion.

With all the research being made with the combination of both natural and synthetic materials, and since it has been proven to create porous and local bioactive environment to regenerate injured tissues. It is probably the route that will be followed in the skin regeneration field.

## 5 Conclusion

Skin appearance is of extreme importance physiologically and psychologically. Since skin tissue is often subject to injury and consequently scarring, it is necessary to develop new alternatives to enhance its healing process, minimize scar formation, and ensure rapid and efficient skin regeneration. Thus, the use of polymer-based biomaterials in wound treatment as wound dressings and regenerative scaffolds is noteworthy.

Natural polymers present excellent features such as biodegradability, biocompatibility, and biological activity. On the other hand, synthetic polymers are biocompatible, present a versatile chemical structure and are easily modified to obtain distinct mechanical properties and degradation rates. In general, these polymers are cost-effective compared to natural polymers. However, synthetic polymers also have disadvantages when compared to natural polymers, namely, higher toxicity and lack of therapeutic advantage as they are biologically inert. However, it is possible to overcome this by coating the polymer with bioactive molecules and or natural polymers, leading to the formation of a composite material with enhanced bioactivity.

This review presents a detailed description of the polymers from natural polymers, synthetic polymers, or a combination of both, and the newer strategies to improve wound healing treatment.
